# Arsenic—Its Application and Use

**Published:** 1862-05

**Authors:** Wm. A. Pease


DENTAL REGISTER OF THE WEST.

VOL. XVI.] MAY, 1862. [NO. 5.

### Original Essays and Communications.

### ARSENIC—ITS APPLICATION AND USE.

BY DR. WM. A. PEASE.

(Read at a meeting of the Mad River Valley Dental Society, April 1st.)

GENTLEMEN—As arsenic has recently been the subject of several articles in the dental journals, and as it is now engaging the attention of the profession, it may not be inappropriate to call your attention to it for a few minutes. In doing so, I do not expect to instruct, and I hardly hope to entertain you, as you all have had a varied experience, and are familiar with its use; yet, trite as the subject is, I am sure of receiving that considerate attention that always characterizes our meetings, and, if I present nothing new, I shall, at least, afford you an opportunity to compare your experience with mine.

Arsenious acid is invaluable as a means for destroying the exposed pulps of teeth. There is no other known article in the Materia Medica that accomplishes this purpose so quickly, so well, with so little injury to the teeth, and so little pain or inconvenience to the patient. Indeed, so necessary and common is its use, it affords a sad commentary on the value people set by their teeth, as it shows that they habitually neglect them until driven to the dentist by actual pain. The daily use of it in all dental offices, often repeatedly in the same mouth within short periods of time, gives it some importance to the toxicologist in Medico—legal investigations, as he by his delicate tests, may, possibly, discover traces of it in a subject, where it had not been used for a criminal purpose.

Arsenic is used in a variety of ways to destroy the pulps of teeth, and it accomplishes it in all, with greater or less facility. It is used in substance, in solution, and combined with other materials. It is applied directly to the exposed part in powder, in paste, or upon a pledget of cotton. By some, it is carefully applied, and as carefully sealed into the hollow of the tooth, by means of wax or other material; and by others, a portion is carried into the cavaity of decay on a dossil of lint; over which a dry piece is packed, or one saturated with a solution of some gum, which they think is a sufficient protection against escape, leaving the patient to remove it at the proper time. All of these methods are available; for a much minuter quantity of arsenic, than is generally used, is sufficient to destroy the pulp; and they are generally safe, because the quantity used is small; it is not easily displaced, and arsenic is sparingly soluable in the juices of the mouth. Notwithstanding this diversity of application by eminent practitioners, who are all successful, it appears to me that there is a choice, and I propose to give my reasons for the same.

As the chief object in destroying the pulp is the preparation of the tooth for a plug, the simple act of distruction is of minor importance compared with the condition in which the tooth is left, as to susceptibility to unfavorable impressions. The destruction may be effected by an escharotic, the cautery, or a probe; but no one at all familiar with the subject would say that they would all leave the tooth in the same condition, alike free from an inclination to take on inflammation. A tooth, whose periosteum has received a shock, however slight, has received a susceptibility that may or may not be developed into periostitis; it is extra-hazardous, and must thereafter be considered a subject for a doubt. It is well known that a tooth in which the pulp has died spontaneously is in a far worse condition than one where it was destroyed; not because it is not possible to remove the pulp or its debris as effectually in the one as the other, but from the condition in which it leaves the periosteum. The preparatory destruction of the pulp too often simulates its spontaneous death; or what is the true cause, its death from accidental chemical or mechanical irritation, which is always preceded by inflammation. To be effectual and safe, its destruction should be quick and sudden, a lingering death is chronic periostitis. It is this which furnishes the key to the excellent results, in the hands of the old practitioners, which followed the use of the probe. The destruction was instantaneous, the pulp very often came away with the probe, and the shock was not sufficient to light up periosteal inflammation. Without intending it, they were unconsciously producing that anatomical and physiological condition which modern science has had to reason out and establish, as necessary, after the disastrous results which followed the use of arsenic had been experienced. Both aimed at the same result—the destruction of the pulp, so that the tooth could be plugged without pain; but in the one case the destruction involved the removal, in the other this was not necessary, and is only now practiced to insure the subsequent health of the parts. From this it will be seen that the manner in which the destruction is effected is important; and that, where the pulp is exposed, our chief concern should be, not how to destroy it, but how to destroy it so as to insure to the parts, at the time and immediately after physiological rest; to give them time to adapt themselves to the altered condition of nourishment and circulation, without shock or engorgement. This can be accomplished at times by all methods, but it can not always be secured by any one; there will occasionally be failures; yet, there is a way of applying the destroying agent which harmonizes with theory, and is successful in practice. Bearing in mind that the destruction should be quick, it will follow, of course, that the fewer impediments there are to the immediate action of the destroying agent, the more successful will be the result; and that, hence, the cavity of decay should be prepared as a preliminary condition.

The cavity being prepared, a portion of arsenic is placed on a slab and thoroughly mixed with creosote. The mixture should not be stiff enough for a paste, nor yet so liquid as to easily drop from the point of a suitable instrument, used to carry it to the place, and deposit it on the exposed pulp. Then, if it is thought necessary, by moving the instrument gently, different ways, it can be so spread out as to touch and come into immediate contact with all the surface exposed. If the cavity is so situated that the point of exposure can not be seen, it can be sufficiently well ascertained, and the preparation applied; after which, by dipping the point of the instrument in creosote containing a saturated solution of arsenic, the surface can be so plastered over as to render it certain that the pulp is covered. Then a piece of softened wax should be quickly applied, to seal in the preparation, before it is wet by the juices of the mouth. If this is done dexterously, and the wax is not pressed so deeply into the cavity as to press upon the pulp, there will be no pain; and if the patient follows the directions, as to eating, he will return the next day, having suffered no pain; and after the wax is removed, the cavity will be found dry and the nerve destroyed. The advantages of this method are that it is simple and effectual; the preparation is confined to the cavity, and there is very little taste from its escape into the mouth, and the percentage of cases where pain is felt, that were not clearly due to mechanical irritation from pressure of the wax, will scarcely amount to an exception. The same method should be employed when morphine is used; but with morphia there will be more pain, because it first acts as a stimulant; and if the small quantity that comes in contact with the pulp exerts any narcotic influence, it is at a time when it is of no value, as the pulp is then nearly destroyed. But, when, for any reason, as in a very large cavity, where the point of exposure can not be ascertained, and it is desirable to spread the preparation over a considerable surface, greater quantity is wanted; morphine can be used, but I prefer any simple powder to gain the necessary bulk—tooth powder answers very well. When we wish to avail ourselves of the narcotic properties of morphine, we should apply it an hour or two before the arsenical preparation; in which case, it may be of some value in certain irritable conditions, but from the trouble and delay it occasions, it will seldom be employed.

This method is preferable to the application on cotton, because, unless the cotton is saturated with a solution, there is not the same certainty that it reaches the exact point of exposure, and even when it does, the contact is not so complete and intimate as when the preparation rests directly upon it; and besides, the solution is not sufficiently strong to act with that instant vigor that destroys at once, without irritation or absorption; a great desideratum to the dentist. But, when applied on cotton, the cotton must be pressed into the cavity to give it some degree of contact with the pulp; and that pressure will cause mechanical irritation and pain; and if that piece of cotton is secured by a dry one, or one saturated with an alcoholic solution of some gum, the presure is generally increased; and still, if instead of securing the first pledget with cotton, wax is substituted, the pressure will be generally increased, and the elasticity of the cotton will loosen the wax. By some practitioners, a solution of gum sandarach is used to saturate a pledget of cotton for a cap, on the principle that the alcohol will be immediately evaporated, and carried away by the juices of the mouth; and that the insoluable gum will remain and form an impervious stopper or cap. A moment's reflection will show that this is objectionable, as the alcohol immediately cuts the creosote and abstracts it from the arsenic; and besides, the quantity of gum is not sufficient to exclude the moisture from the cavity. They thus gain a poor stopper, at the expense of an important ingredient of the preparation they wish to retain. Perhaps they were led to attach an undue importance to this gum as a stopper, from the absence of all taste of creosote, which was destroyed by the alcohol. In the destruction of the pulp, creosote plays an important part. An active and excellent escharotic, it instantly cuts through the endo-osteum, carrying with it the arsenic, to act with vigor on the denuded surface. It by this means allays pain, by removing tension in an inflamed pulp, in the same manner, but without the pain the puncture of an instrument would produce. As used in excess, it forms a carbolate of arsenic, which, as far as our present knowledge goes, is not readily absorbed. It seems to act by rapidly destroying tissue, or vitality, and retarding decomposition; and there is reason to believe that it will be found a better preparation for topical dressings for foul ulcers, the destruction of cancerous or fungus growths, than other preparations of the same metal.

Growing out of and intimately connected with this subject is an examination of the subsequent treatment and condition of the pulp, in order to discover the relative merits of this or any other mode of application. After the destruction has been effected, the question arises at what time should the pulp be removed from the tooth—whether immediately or after a time? If it is deferred, how long should it be to secure the most favorable results, and what are the *post-mortem* conditions as revealed after removal, or after extracting and splitting a tooth, that enable us to determine the proper time. Philosophical reasons founded upon a knowledge of the nature of the pulp are not sufficient, unless supported by experience. What are the facts? Taking into consideration the known properties of the destroying agent, and the favorable condition of the pulp, as to warmth and moisture, for early and rapid decomposition, it would be supposed that softening or decomposition would take place later in a pulp destroyed than in one that had died spontaneously or from inflammation; and as we have chiefly to deal with these, a knowledge of the action of the destroying agent, as it effects the following points, becomes of practical importance.

1. Absence of pain to the patient.
2. Facility of removal of the entire pulp from the tooth.
3. Absence of shock.
4. Physiological rest.
5. Absence of bleeding or exudation.

Choice can not always govern the operations of the dentist. Some regard must be paid to circumstances, and the necessities of the patient; but, where they are favorable, some rule should be adopted, founded upon actual knowledge and experience. While I adopt a mixed practice, sometimes plugging the tooth the day after the preparation was applied; those cases are exceptional, and they are not the ones from which I expect the most favorable results. The pulp is the organ that largely contributes to the nourishment of the tooth; having nerve, artery and vein. The nerve sends filaments, or conductors of sensation, to all parts of the dentine; and all are supplied with nutrition from the artery, as well as a certain amount of increase of substance or density; and there is reason to believe that there is inter-communication and circulation between the endo-osteum and periosteum, although their functions are independent; the pulp presiding over the nutrition of dentine, and the periosteum over that of the CRUSTA PETROSA; having for its object the maintenance of the tooth in its place, and a vital connection with the system, after the pulp is destroyed. How, then, and at what time is this important organ to be removed from the tooth, to insure the most satisfactory results, as measured by the conditions enumerated above. The pulp is so highly organized, that after it has been deadened, its circulation and its vital connection with the system destroyed; or, in other words, after that condition has been superinduced from which decomposition must take place, after it has already commenced, as shown by a change of color to that of extravasated blood; it is still very often sensitive to the touch of an instrument, and this sensitiveness will continue for several days; although it seems to be peripheral, as after a slight puncture or penetration below the investing membrane, it generally disappears.

Just before the commencement of decomposition, varying from the sixth to the tenth day, certain phenomena frequently appear, vexatious to the patient and practitioner, that may lead to grave errors in diagnosis. They consist in a hypersensitiveness to heat or cold, sometimes to both. Unless the patient is prepared to expect this, he will frequently lose confidence in the operation, and at times go to another dentist. I have known of several cases where the pulp was repeatedly dosed for this condition without benefit. Indeed, a differential diagnosis is sometimes difficult, as the exaltation of sensation is as great to touch as to change of temperature. The remedy consists in a puncture; and when the pulp was punctured at the time the preparation was removed, this condition rarely happens. It would be interesting to refer to some other changes through which the pulp passes, but time will not admit. At a period of time varying from one week to ten days after deadening the pulp, sensitiveness disappears; decomposition is commencing, and the pulp loses its attachment to the walls of the tooth. It is then in a good condition to remove, as it is not sufficiently softened to tear, and it generally comes away entire, the first trial. But, even at this time, when it comes away entire, there is generally quite a sharp pain, arising from a mechanical cause. As the instrument passes down into the pulp, generally to a considerable depth, the patient sits quiet and unconscious; it is then made fast and gradually withdrawn, and the parting at the foramen is distinctly felt; still, the patient is unconscious or but slightly uneasy. As the instrument is further withdrawn, it drags the pulp back upon itself, and there is a partial or complete intussusception, which so increases the bulk or density at the point seized, that, as it is further withdrawn, it acts like the piston of a syringe, and makes so great a vacuum as to draw upon the parts beyond the foramen and cause pain. If we carefully examine the pulp after its removal, to see if we can discover, from its physical condition, anything that will enable us to determine the proper time for that operation, I think you will agree with me that there is unmistakable evidence that it should not be immediate. At the point of exposure it is of a dark purple color, at times shaded with red, varying with the time of examination, and from other causes. This color gradually changes as we approach the foramen, often, at a point about one-fourth or eighth of an inch distant it is of a dark red—ASTRO SANGUINEA. Here the discoloration generally terminates abruptly in a well defined line or margin, strongly contrasting with the unchanged color of the remaining portion, which is natural, but bloodless, and cadaverous. So universal is this condition that I do not now recollect in several years any material departure from it. From such POST MORTEM examinations, I think we can deduce the following considerations, that will go far to establish the propositions I have before enumerated; and, perhaps, settle the dispute as to the action of arsenic on intra-dental tissue. They show it is not absorbed, or rather that it does not pass the foramen, and consequently that it adds nothing to the difficulties we have to contend with, viz: those resulting from severance of the parts—a simple wound and that under favorable circumstances. But, if the time I have indicated be allowed to elapse, there is no wound; for nature has anticipated the operation and prepared for the separation, the same as she does after the ligature of a polypus. There may have been, for a time, a slight vascular fullness, but it could not be great from this cause alone, as the quantity of blood that passes the foramen is so small as to be easily removed by anastomosis. Thus there has been a period of physiological rest; the circulation has been provided for and equalized; and if the location was favorable, the pulp would be cast off and it would fall away. There is no shock, no wound, no flooding with blood, no exudation, and the tooth is in a favorable condition for the plug; which should be inserted immediately. And here let me call your attention to a matter not exactly germane to the application and use of arsenic, but one, nevertheless, that grows out of its use, and is essential to the subsequent health of the tooth. I refer to the manner in which the pulp is removed, and the tooth plugged. The great object for the removal of the pulp from the canal is to escape irritation, but no advantage is gained if we remove one irritating agent and substitute another—the saliva. Hence the manner of removal and the manner of plugging the tooth is important.

The following method can be recommended:—The cavity of decay is first prepared by freeing it from all extraneous matter and diseased bone; the passage to the pulp canal or chamber is next enlarged sufficiently to give easy exit to the pulp on the broach. Having the pluggers, the gold for the canal and everything in readiness, the napkins are adjusted, the cavity of decay dried, the pulp is withdrawn, and a piece of gold is carried to its place in the canal so as to exclude all saliva, if there is need of haste; but, in general, the canal should be completely filled at once, and the cavity of decay, if advisable. By this means the teeth of the Inferior Maxilla can be as successfully plugged as those of the Superior.

Having noticed the most important modes of application and uses of arsenic for the destruction of intra-dental tissue one point remains for consideration. In some few cases the pulp is not entirely destroyed by the first application or rather sensation remains to such a degree the patient will not allow the pulp to be removed, and it is inconvenient to let the case rest till sensation subsides. In such cases it is well to apply a little creosote, holding arsenic in solution, to the part and let it rest as long as possible, or a day or two. But a more troublesome class of cases arises from the parting of the pulp just below the margin of the gum, leaving the remaining portion angry and sensitive. This in general arises from a too early attempt to withdraw it, and sometimes, from a deposit of OSTEO-DENTINE at the place, which encroached upon and weakened the pulp.

After a deal of trouble with such cases and irritable patients, I have found it convenient to insert a temporary filling and let the tooth rest. About eight years since the pulp parted nearly one-fourth of an inch below the margin of the gum in a central incisor. The tooth was worn away by antagonism and the cavity was nearly on the cutting edge. The gentleman was exceedingly sensitive and refused to let me touch it further. Desirous of saving the tooth I carried a portion of arsenic to the place and capped it in with wax. Everything was favorable, the cavity was accessible, the preparation was well retained until removed the next day, when a dry piece of cotton was placed in the canal—directed to return in a few days. He returned and complained that something was wrong with his gum. It was inflamed, and a wash was applied—to return. The next examination I found an annular piece of the socket had exfoliated at the point where the pulp parted, which was removed. The gum settled down to the tooth, the irritation subsided, and as the tooth still had some firmness it was allowed to remain. The subsequent history of the case I am unable to give, as the gentleman left the city soon after. The tooth was however in his mouth nearly two years later. Here was a case of absorption through the dentine and resulting necrosis of the socket. It is interesting as showing the anatomy of the parts and also the danger of such applications. It may never happen again, and such cases, without care, may be too frequent.

Having passed in review the application and use of arsenic for the destruction of the dental pulp, the next important use of it consists in the destruction of sensitiveness of dentine. Time will not admit of an extended inquiry into the pathology of this affection, the views of different writers concerning it, or the means they consider most suitable for its removal. It must suffice for the present to say that it arises from the same cause as odontitis, viz: chemical or mechanical irritation and that it may be removed by the same means. After the loss of the enamel the peripheral filaments of the pulp are liable to inflammation or destruction in the same manner as the pulp after exposure. As an exposed pulp may remain for considerable periods without irritation, or it may quietly die without ever having caused disturbance; so its minute filaments may pass through the same conditions like it, or they may give abundant trouble. While I avoid as much as possible all treatment of this affection, preferring, if at all bearable, to prepare and plug the tooth at one sitting, I do not hesitate to deaden the sensibility, when necessary, subject to some conditions.

1st. It is not indicated and it is of doubtful propriety in young subjects, except in cases of superficial decay.

2nd. The same is true of older persons where the dentine is soft and porous.

3rd. It should not be resorted to when but a thin septum of bone separates the cavity of decay from the pulp.

As the object of the application is to produce a superficial result the preparation should be concentrated in order to accomplish it in the shortest possible time. For this reason the same preparation should be used as that for destroying the pulp, because it comes into more immediate and intimate contact with the tender surface than in any other form.

The objection to the combination with creosote, as urged by some, that it is more liable to be absorbed and discolor the tooth is not founded on fact; for a tooth is never discolored so long as the pulp is alive. If discoloration take place the indications are palpable that the canal should be instantly opened and evacuated of its contents; and if periostitis is not present the fang should be filled nearly to the margin of the gum; the balance left open, when it will generally disappear. In forming the prognosis of any given case after due attention has been given to the unfavorable conditions enumerated before the question must be decided what will be best for the patient under the circumstances—whether to proceed to destroy the sensitiveness and take the chances of a dead pulp, of discoloration and the unfavorable conditions as to the future health of the tooth, or to purposely expose and destroy the pulp, which can often be done with but little pain, and treat the tooth as herein before described. When it is a simple case of denudation of enamel, the most sensitive and troublesome, I should proceed to destroy sensation with the strongest preparation I could make and in the quickest possible way; keeping the patient by me for a time for an occasional examination in order to plug at the first practicable moment. In this way sensation may often be deadened in from twenty minutes to an hour so as to render the operation bearable; and then no apprehension need be felt for an untoward result. The danger lies in exposing the tooth to the action of a small quantity or a weak preparation of arsenic for a long time.

There remains but one other condition where arsenic is indicated for dental purposes—the destruction of fungoid growths of the pulp or gum. When accessible such growths of the gum can generally be removed to the best advantage with the lancet, as bleeding is easily controlled and in others creosote, which is innoxious, answers very well. It can be reapplied if necessary. In cases of fungoid-growths of the pulp creosote holding arsenic in solution is indicated, and it may also be used to destrcy similar growths of the gum that lap over and into large cavities when the pulp is dead; as it acts with a little more vigor than creosote alone; but it should never be used when it will unneccssarily occasion the death of the pulp. The topical use of arsenic for these or other purposes should be governed by the same rules and precautions as when applied to other parts of the system.

